# Prenatal diagnosis of orofacial clefts: unveiling the parents’ experience

**DOI:** 10.1590/1984-0462/2023/41/2022004

**Published:** 2023-01-20

**Authors:** Verônica Aparecida Pezzato da Silva, Marina Gifalli, Francine Aroteia Capone, Francely Tineli Farinha, Priscila Capelato Prado, Armando dos Santos Trettene

**Affiliations:** a Universidade de São Paulo, Bauru, SP, Brazil.

**Keywords:** Cleft lip, Cleft palate, Prenatal diagnosis, Qualitative research, Pregnancy, Parents, Fenda labial, Fissura palatina, Diagnóstico pré-natal, Pesquisa qualitativa, Gravidez, Pais

## Abstract

**Objective:**

To understand the experience of parents regarding prenatal diagnosis of orofacial cleft in their children.

**Methods:**

Descriptive study with a qualitative approach, carried out in a Brazilian public tertiary hospital between January and March 2019. Parents who were accompanying their children during hospitalization for primary surgeries and who had received the diagnosis of malformation during pregnancy were included in this study. Data was collected through semi-structured interviews, which were audio-recorded and transcribed in full. To prepare the results, Content Analysis was used in the Thematic modality.

**Results:**

The sample had 17 participants: 16 mothers and one father. From the speeches, three categories were unveiled: dealing with the unknown, assimilating the diagnosis, and positive and negative implications of prenatal diagnosis.

**Conclusions:**

We learned how complex and conflicting it was for parents to receive the diagnosis of malformation in their children, and that family and professional support was essential to the process of assimilation and coping. The findings point to the need for planning and implementing interventions, protocols and/or public policies aimed at assisting these parents in this period.

## RESUMO

**Objetivo:**

Compreender a experiência de pais quanto ao diagnóstico pré-natal da fissura orofacial em seu filho.

**Métodos:**

Estudo descritivo, de abordagem qualitativa, realizado em hospital público e terciário brasileiro, entre janeiro e março de 2019. Foram incluídos pais que acompanhavam os filhos durante a internação para realização de cirurgias primárias, e que haviam recebido o diagnóstico da malformação durante o período gestacional. A coleta de dados foi realizada por meio de entrevista semiestruturada, que foi gravada e transcrita na íntegra. Para confecção dos resultados utilizou-se a Análise de Conteúdo na modalidade temática.

**Resultados:**

A amostra constou de 17 participantes, dos quais 16 mães e um pai. Com base nos discursos, desvelaram-se três categorias: lidando com o desconhecido; assimilando o diagnóstico; e implicações positivas e negativas do diagnóstico no pré-natal.

**Conclusões:**

Apreendeu-se quão complexo e conflitante foi para os pais receber o diagnóstico da malformação em seu filho, e o apoio familiar e profissional estabeleceu-se como indispensável ao processo de assimilação e enfrentamento. Os achados apontaram a necessidade de planejar e implementar intervenções, protocolos e/ou políticas públicas, para assistir esses pais nesse período.

## INTRODUCTION

Orofacial clefts are the most common malformations that affect the face, being th result of a failure in the fusion of the structural process of the lip and/or palate, which may be unilateral, median or bilateral. Cleft lip and alveolar ridges occur in the first trimester of pregnancy, up to the 8th week, while cleft palates occur up to the 12th week. The incidence in Brazil is of 1/650 live births, and the etiology is multifactorial, related to genetic and environmental aspects.^1–3^

Although prenatal diagnosis of orofacial clefts is possible by ultrasound, it is necessary to visualize the malformation. In fact, only after birth is it possible to accurately assess the extent of the malformation and its functional implications.^4,5^ In association with this, the woman experiences numerous emotional and physiological transformations during pregnancy. Also during this period, parents build the idealization of a perfect and healthy baby, so when they learn they will have a child with malformation, a long-awaited and desired moment becomes painful, bringing negative and conflicting feelings.^6–10^

In this sense, the assimilation process is slow, and difficulties add up to the lack of adequate information, making the benefits of prenatal malformation diagnosis questionable.^11,12^

In short, the complexity experienced by parents when they receive the diagnosis of orofacial clefts in their children is evident. Given the above, we sought to answer the following question: how was it for parents to learn about the diagnosis of their child's orofacial cleft during pregnancy? By understanding this experience, we hope to contribute to the planning and establishment of interventions aimed for these parents to favor the process of assimilating the diagnosis, their physical and mental health, and to prepare to care for a baby with this special condition. Thus, the objective of this study was to try and understand the experience of parents when it comes to prenatal diagnosis of orofacial cleft in their children.

## METHOD

This is a descriptive study with qualitative approach, guided by the Consolidated Criteria for Reporting Qualitative Research (COREQ).^13^ The institution chosen for the research was a Brazilian public tertiary hospital, reference in the care of patients with craniofacial anomalies and related syndromes, located within the state of São Paulo.

Parents of infants with orofacial clefts who were accompanying their children during the postoperative hospitalization of primary cheiloplasty and/or palatoplasty surgeries, whose diagnosis had occurred in the prenatal period, were invited to participate. Parents of infants with syndromes and anomalies other than orofacial cleft were excluded. The intentional and convenience sample was defined by theoretical saturation^14^ in the 17th interview. Seventeen fathers participated, being 16 mothers and one father.

Previously, the participants were approached by the researchers and invited to participate, while the objectives and implications of the study in clinical practice were clarified. Data was collected between January and March 2019 through a semi-structured interview, which was recorded and transcribed in full. The triggering element was: how was it for you to find out about the diagnosis of your child's orofacial cleft during prenatal care? The average duration of the interviews was 20 minutes, and an individual approach in a private environment.

At the end of each interview, a recording was presented to the participant, and they were asked to add or change anything. No modifications or new approaches were needed. Data collection was carried out exclusively by the main researcher, who received previous training, and by her supervisor, who has experience in quantitative design studies. It is noteworthy that both are nurses and did not work in the unit, that is, they did not have direct contact with the participants.

After transcribing the speeches for qualitative analysis, the results were treated by inferring and interpreting the contents by categories and similarity, following the methodology of Content Analysis in the thematic modality, with the following steps: pre-analysis, material exploration and interpretation. Thus, texts were read in order to apprehend the core meaning of the concept, from which the coding categories emerged, and, later, the units that represented a real meaning were made to respond to the proposed objectives. in the study. The semantic criterion of words was used to categorize them, aiming to bring the real meaning of the registration units and, finally, the treatment of results was carried out by inference and interpretation of contents, resuming the reasoning and justification of the study, basing the correct sense of the analysis.^14^ At the end of the study, the results were presented to the parents when they returned for care at the institution where the research was carried out.

The research received a favorable opinion from the Research Ethics Committee involving human beings of the institution, through CAAE: 02759718.5.0000.5441, and all ethical precepts were complied with. To identify the speeches and ensure anonymity, the letter “P” of participant was used, plus sequential Arabic numbers.

## RESULTS

At first, 35 parents were approached consecutively. Of these, six only learned about the malformation at the birth of their children, and eight chose not to participate in the study. Among the remaining 21, the children had other malformations associated with orofacial cleft in four cases and were excluded from the study. Finally, following the methodology proposed in this study, 17 parents participated, being 16 mothers and one father. The mean age of participants was 26 years old, most of them had two children, were in a stable union and had with an employment relationship. None of them had previous knowledge about family history of orofacial clefts.

Three categories emerged from their speeches:

1. dealing with the unknown;
2. assimilating the diagnosis; and
3. positive and negative implications of prenatal diagnosis.

### Dealing with the unknown

A range of negative feelings was perceived, including shock, guilt, despair, anger and fear, all related to the idea that something had gone wrong during pregnancy to cause the malformation, not knowing how to take care of the child, and the reaction of the spouse and family members.

*“I'm a smoker […] I wondered if it wasn't what caused the malformation […] I feel guilty seeing her going through this. Also, I feared not knowing how to take care of her.” (P7) “I was afraid of people's judgment […], thinking that he was born this way because of his parents’ fault or carelessness, […] as if we had caused this problem.” (P13)*

*“I was desperate, I researched about treatments, medicines that could reverse the situation […] I was angry with God! We go through so much in this life […] I didn't even want to get pregnant, […] and now I have a baby with a problem like this, apart from the problems we already have.” (P2)*

*“I found out and kept it to myself […], I didn't tell anyone […] I hid it until the end of the pregnancy. The father found out at the time of delivery and was shocked when he saw the baby. I didn't know what his reaction would be, or of the relatives […] I preferred to suffer alone.” (P8)*

The parents were found to be concerned with functional aspects related to the malformation, such as eating and speaking.

*“I thought about what his diet would be like […] I feared he wouldn't be able to feed from the breast and bottle. My fear was that he would be tube fed.” (P13)*

*“I cried all the time […] I imagined that he would not be able to breastfeed […] Another concern was speech.” (P5)*

The fear of the child having other anomalies associated with the cleft was also expressed.

*“I was worried them being born with a syndrome […]. I know that the cleft can be accompanied by a syndrome.” (P10) “I was afraid that she would have associated diseases: heart, mental, other anomalies in her feet, hands […] I also fear people's prejudice.” (P12)*

Another theme brought to light was related to the psychosocial context, including prejudice and bullying, that is, problems related to esthetics and socialization, in addition to concerns about surgeries.

*“I was worried about the surgeries he will have […], what the recovery would be like […] I fear he will suffer prejudice or bullying when he goes to school.” (P15)*

*“I thought about how I was going to go out on the street with him […], what people would say […], what it would be like to have a baby like that! I was worried about bullying.” (P14)*

*“I was worried about the bullying she could suffer at school when she gets older […] I want her to have the surgeries soon and be fine as to esthetics.” (P2)*

### Assimilating the diagnosis

After the initial phase, the process of assimilation by parents and/or family members took place.

*“My family is very close […] when I told them what I heard that this (cleft) is just a detail, that love would be double and that anything we needed we could count on them, especially my mother and my brother. That helped a lot!” (P4)*

*“My husband was stronger than me […] he tried to find out what the treatment was like, how to take care […] but always asking if I was okay, saying that I shouldn't worry, that God was with me. us.” (P5)*

For some participants, the assimilation process was favored by contact with other parents and family members experiencing the same situation.

*“We came to the hospital to meet some parents and children who had this problem […] we had little knowledge about the subject […], the nurses helped us a lot.” (P11)*

*“I searched the internet for centers that care for children with clefts and found Centrinho (hospital) […] I came for an appointment and the experience was very positive […] we became more relaxed and confident.” (P17)*

*“The service lasted two hours […] it was possible to clarify many doubts about feeding, surgeries and even prejudices […] I recommend this service to all parents who go through this situation.” (P1)*

### Positive and negative implications of prenatal diagnosis

The positive aspects prevailed. Examples include: preparation for the care of the child after birth, knowledge about orofacial clefts and psychological support.

*“We wanted to find out if the baby could be breastfed, if he would need a specific bottle, what the cleft was, why the cleft was formed, if it was genetic, if it was a lack of vitamins, find out at the hospital that I was going to receive it, how they would receive the baby, if they were ready.” (P4) “I think it's better to learn the diagnosis during pregnancy because we can prepare ourselves […] to research the subject and not have that shock at birth.” (P9)*

*“I had never seen a cleft and didn't even know what it was. I think it's important to know during pregnancy, because knowing only at birth makes the shock greater. I think that if one knows it beforehand, one can prepare emotionally […]. I was followed up by a psychologist, which helped me a lot.” (P8)*

However, some negative aspects also emerged from the speeches, including: anxiety until birth, lack of support from qualified professionals and confusing and sensationalist information obtained on the internet.

*“Even though I knew about the cleft during pregnancy, I did not have a professional guide me […]. I was bewildered and did not know what to do. For me, knowing about the cleft before the baby was born only brought anxiety and worry. I spent the entire pregnancy like that […], along with my husband and my whole family.” (P7)*

*“Finding out before or after the baby is born is just as difficult […]. Knowing beforehand didn't help much […] Finding out beforehand makes us more anxious, thinking about what we're going to do after the child is born.” (P2) “We did the ultrasound and the doctor told me about the cleft. I made the mistake of searching the internet, and I got more confused. I think that if I didn't know before birth it would be better.” (P5)*

## DISCUSSION

At the time of discovering the pregnancy, parents idealize a healthy and perfect baby and when they receive the diagnosis of a child with orofacial cleft, they manifest feelings, actions and reactions including: shock, guilt, despair, anger and fear.^15,16^ In fact, in the present study, negative feelings and psychosocial aspects were expressive, which can result in significant disruptions for caregivers and family members.^7,12,15^ In this sense, it is up to health professionals to emphasize the positive aspects of the child and not address only the limitations.^17,18^

Some parents reported fear of sharing the diagnosis of the malformation with their spouses and/or family members, fearing negative reactions such as disdain and denial. The acceptance process is linked to the family profile, social and economic contexts, previous cases in the family, whether the child is the firstborn, and the severity of the malformation. Thus, support is essential, as parents are encouraged to deal with their feelings and behaviors when coping and searching for information.^6,18–20^

From the diagnosis of orofacial cleft in pregnancy, parents should receive information regarding child care and treatment, as well as psychological support aimed at comfort and emotional backing, both from the family and the health care team.^7,16,17,21^ In general, questions refer to feeding, hygiene, surgical protocol, postoperative care, uncertainties about what children can experience functionally and psychosocially.^16,18^

In this study, concerns related to the functional implications of the malformation were pointed out, especially diet. Food should be offered orally from birth, as sucking and swallowing reflexes are preserved. In addition, methods used should be as simple as possible. Direct breastfeeding is possible, particularly in cases of less anatomical complexity, although constant monitoring of weight gain is necessary.^21^

Another concern expressed by parents was the child having other anomalies associated with orofacial clefts. Between 30 and 40% of children have this malformation associated with other deformities and/or syndromes.^1–3^ In this sense, it is important to investigate other malformations upon morphological ultrasonography, and to request, whenever possible, the fetal karyotype test to aid in diagnosis.^5^

Among the associations, the Robin Sequence stands out, which is characterized by the presence of micrognathia, glossoptosis and, usually, posterior cleft palate. These infants have breathing and feeding difficulties.^22,23^

Another theme highlighted was the concern with the psychosocial context, corroborating the literature.^6,16,18^ In fact, patients with clefts can present psychosocial problems related to prejudice, bullying and esthetics. In this sense, another study pointed out that parents want to learn about the best treatment options to minimize the possibility of their children experiencing social stigma.^15^ However, the manifestation of these problems will depend on how they will face the difficulties and coping modalities.^24,25^ These children are submitted to primary surgeries for anatomical and esthetic correction, although the treatment extends to adulthood.^2,26^

The positive aspects of prenatal diagnosis of orofacial cleft were frequent and are related to getting prepared for child care, acquiring knowledge about the orofacial cleft and receiving psychological support. Once they learn about the malformation, the family will have time to adapt, that is, prenatal diagnosis allows for adequate and timely parents counseling.^16,27^

In this sense, a North American investigation pointed out that mothers of babies with orofacial clefts who did not receive a prenatal diagnosis had higher scores for postpartum depression, anxiety and incidence of fear, which indicates that prenatal diagnosis can contribute to a positive adaptations of the mother in the postpartum period.^27^ As soon as the malformation is discovered, parents should be immediately referred to a multidisciplinary team for counseling and support, considering the repercussions on the quality of life of parents and family members.^28,29^

It is also important to highlight the need to seek specialized centers that provide qualified, humanized care by trained professionals. In the institution where this research was carried out, care through nursing consultations for pregnant women, parents and family members whose child was diagnosed with orofacial cleft was shown to be promising, being identified as relevant even among the participants of this study. Parents had the opportunity to resolve doubts and receive guidance from professionals specializing in the rehabilitation process. After care, parents report being less anxious, more secure, confident and hopeful.^16^

On the other hand, negative aspects were also apprehended, including anxiety until birth, lack of support from qualified professionals and confusing or sensationalist information obtained on the internet. Indeed, in prenatal care, parents and family members need humanized and enlightening care as to minimize or resolve doubts and questions.^30^ In practice, some parents who received prenatal diagnosis experienced more suffering when health professionals were unable to answer questions,^19^ which highlighted the need for them to be attentive and prepared when providing care to parents and family members in this situation.

Parents seek to understand what could have been the cause of the malformation in their baby, often resorting to internet searches, imagining that they will know the reason why their child was born with that deformity. However, seeking information from unreliable sources increases stress and insecurity.^16^

Some strategies are pointed out as facilitators of the process of diagnosis assimilation by parents and family members—for example, the way in which health professionals inform the diagnosis—, because they affect the parents’ perception of the malformation and how they will deal with the challenges. Psychological and social support is essential for processing emotions, promoting the use of coping skills, and addressing concerns about social stigmatization and the psychological impact of cravings, as well as ongoing team support for parents and family members.^6,20^

Regardless of when the diagnosis is made, in addition to receiving guidance on treatment, parents should have the opportunity to interact with other couples with similar experience, participate in courses for parents, as these are contingencies that favor coping, even when it comes to marital relationships, usually weakened by this experienced.^9–18^

Finally, the data being collected in a single interview may not have been enough to capture all the phenomena involved in the process of assimilating prenatal diagnosis of orofacial clefts, which constitutes a limitation. Furthermore, the expressive participation of mothers dd not allow for a perception of the fathers or other family members in a broader way, so these are themes to be addressed in future studies.

In summary, we could see how complex and conflicting it is for parents to receive the prenatal diagnosis of orofacial cleft in their children. Therefore, interventions, protocols and/or public policies aimed at assisting these parents in this period should be planned and implemented.
